# The Impact of Tumor Elongation on Facial Nerve Outcome after Surgery for Koos Grade 3 and 4 Vestibular Schwannomas in the Semi-Sitting Position via the Retrosigmoid Approach

**DOI:** 10.3390/jcm13175319

**Published:** 2024-09-08

**Authors:** Franziska Glieme, Lisa Haddad, Felix Arlt, Martin Vychopen, Clemens Seidel, Alonso Barrantes-Freer, Erdem Güresir, Johannes Wach

**Affiliations:** 1 Department of Neurosurgery, University Hospital Leipzig, University of Leipzig, 04103 Leipzig, Germany; lisa.haddad@medizin.uni-leipzig.de (L.H.); felix.arlt@medizin.uni-leipzig.de (F.A.); martin.vychopen@medizin.uni-leipzig.de (M.V.); erdem.gueresir@medizin.uni-leipzig.de (E.G.); johannes.wach@medizin.uni-leipzig.de (J.W.); 2 Comprehensive Cancer Center Central Germany, Partner Site Leipzig, 04103 Leipzig, Germany; clemens.seidel@medizin.uni-leipzig.de (C.S.); alonso.barrantes-freer@medizin.uni-leipzig.de (A.B.-F.); 3 Department of Radiation Oncology, University Hospital Leipzig, University of Leipzig, 04103 Leipzig, Germany; 4 Department of Neuropathology, University Hospital Leipzig, University of Leipzig, 04103 Leipzig, Germany

**Keywords:** vestibular schwannoma, facial nerve, tumor elongation, progression, shape

## Abstract

**Background:** Facial nerve paralysis is a severe dysfunction after vestibular schwannoma (VS) surgery. **Methods:** This monocentric study analyzed 61 patients who underwent sporadic VS surgery in a standardized manner. The primary endpoint was the facial nerve outcome (FNO) at 3 months after VS surgery. FNO was dichotomized into “good” (House–Brackmann (HB) score ≤ 2) and “poor” (HB > 2). **Results:** Poor FNO was observed in 11 patients (18.0%) at 3 months after VS surgery. Radiomic tumor shape features were analyzed, and the AUC of elongation in the prediction of a poor HB at 3 months was 0.70 (95% CI: 0.56–0.85, *p* = 0.03) and the optimum threshold value (≤/>0.35) yielded a sensitivity and specificity of 64.0% and 75.4%, respectively. Multivariable logistic regression analyses considering the extent of resection (</≥93.4%), preoperative tumor volume (</≥2.6 cm^3^), age (</≥55), sex (female/male), and elongation (≤/>0.35) revealed that more elongated VSs (≤0.35; OR: 5.8; 95%CI: 1.2–28.2; *p* = 0.03) and those with an increased EoR (≥93.4%; OR: 6.5; 95%CI: 1.0–42.5; *p* = 0.05) are independently associated with poorer FNO at 3 months after surgery. **Conclusions:** Highly elongated VS shape seems to be a risk factor for worsened facial nerve outcome at 3 months after surgery for Koos grade 3 and 4 tumors.

## 1. Introduction

Vestibular schwannoma (VS) is, with 75% of all tumors, the most common one in the cerebellopontine angle [1]. As a benign tumor, the impairment takes place in the loss of functional hearing and with increasing size in the compression of adjacent structures like the brainstem and facial nerve. Gross total resection (GTR) is suggested as the most effective strategy to achieve long-term tumor control [2]. However, studies have also suggested that a subtotal resection (STR) leads to comparable progression-free survival [3,4]. Because of the benign nature of the VS, preoperative mortality should be as low as possible. Currently, not mortality but low morbidity should be the claim of medical care. Accordingly, different therapy regimes like watch and wait, radiotherapy, radiosurgery, and surgical resection are applied in practice [5,6]. However, especially, larger tumors that compress the brainstem need surgical debulking. Due to the larger tumor size, there is even less space for manipulation, which impedes low morbidity. Therefore, studies suggest subtotal resection (STR) or near-total resection (NTR) against gross total resection (GTR) to provide a better outcome, especially in relation to the facial nerve outcome [7,8,9]. On the other hand, after STR, VS seems to have higher regrow rates [10]. VS shows a large variety in the evolution of growth in volume and shape. It has been shown that shape and texture features may have an impact on predicting VS enlargement after stereotactic radiosurgery (SRS) [11]. While preventing a tumor progression by a radical resection, the preservation of the facial nerve function has a significant impact on the quality of life of the patients. Eye irritations and tear dysfunctions due to peripheral facial nerve palsy lead to necessary follow-up treatments to attain a disability-free life. Therefore, the major priority of the decision-making process of the treatment regime must be the protection of the facial nerve function. As the inflammatory rate is described as an indicator of tumor growth [12], it seems also that the degree of inflammation measured by the MIB-1 index predicts worse long-term facial nerve outcomes in VS surgery [7,13,14]. However, the importance of tumor volume and its individual shape measured in tumor elongation for facial nerve outcome is unknown.

Against this backdrop, the present investigation analyzes the role of tumor shape in sporadic VS patients who underwent surgery in the semi-sitting position via the retrosigmoid approach. The present study is the first one concerning the impact of tumor elongation on facial nerve outcome after surgery for Koos grades 3 and 4.

## 2. Materials and Methods

### 2.1. Study Design and Patient Characteristics

Between January 2012 and December 2022, 120 patients with sporadic VS underwent surgery at the authors’ institution. A total of 61 patients were included in the retrospective analysis. The inclusion criteria were a primary tumor, Koos grade 3 or 4, and available imaging data for radiomic analysis. The patients with neurofibromatosis type 2-associated schwannoma or those who underwent prior radiotherapy were excluded due to the different neuropathology and proliferative potential [15,16]. An interdisciplinary neuro-oncological board consisting of senior experts in the fields of neurosurgery, radiotherapy, neuroradiology, and neuro-oncology was included in the treatment decision-making process. After postoperative CT imaging, the first regular follow-up MR took place 3 months after surgery, and further imaging was on an annual basis. After 3 years of progression-free survival, the control period was extended to 2 years. The facial nerve outcome 3 months post-VS surgery was categorized into “good” (House–Brackmann (HB) score ≤ 2) and “poor” >2 HB [7].

### 2.2. Data Recording

These general preoperative patient characteristics were recorded: age, sex, Koos grade, facial nerve functioning, neurological deficits, and postoperative follow-up data and entered into a computerized dataset (SPSS, Version 29 for Windows, IBM Corp., Armonk, NY, USA). The extent of resection was objectively measured using the volumetric measurement of tumor portions in T1-Gadoliunium-enhanced MR-weighted images and calculated using the following formula: (preoperative tumor volume − postoperative tumor volume)/preoperative tumor volume).

**Figure 1:**
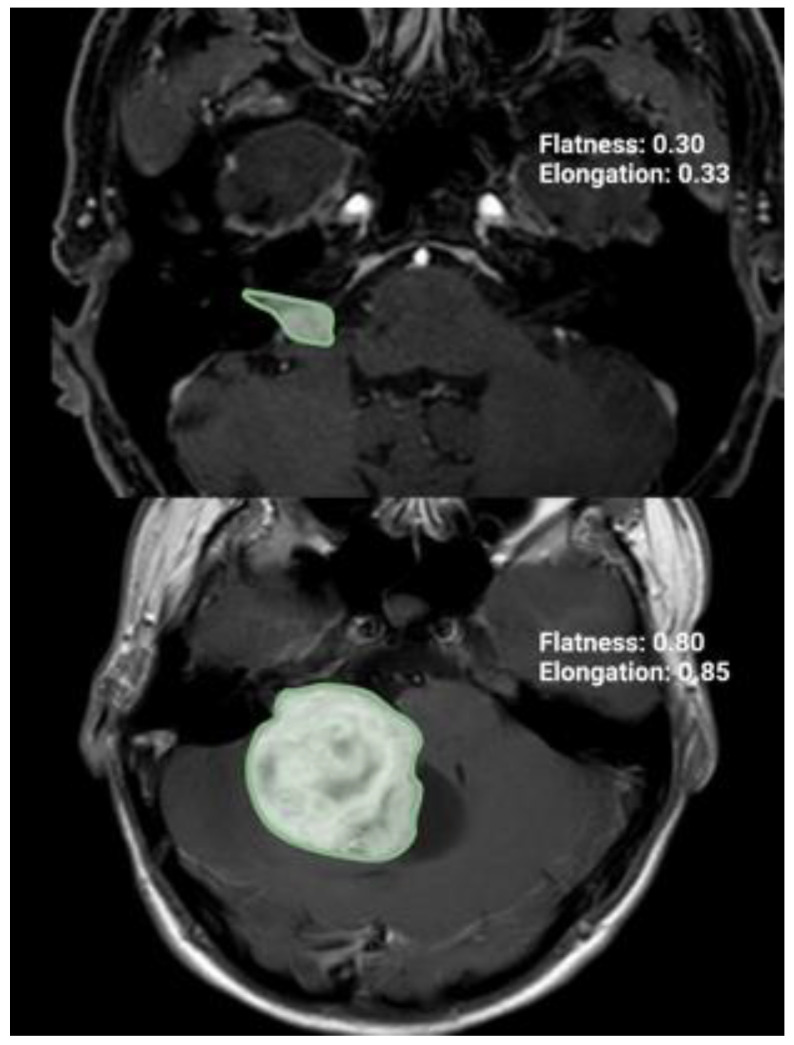
Illustration of two axial T1-Gd-enhanced MR images representing two cases with different radiomic shape parameter elongation.

GTR refers to the complete removal of the tumor, while NTR, STR, and partial resections PR are defined as removing more than 90–100%, 80–90%, and 80% or less of the initial preoperative tumor volume, respectively [17]. Through these MR images, the pre- and postoperative tumor volumes, surface area, extent of resection (%), and radiomic tumor shape features (elongation, flatness, sphericity) were calculated by using volumetric analysis in a 3D slicer software (version 5.4.0, Surgical Planning Laboratory, Harvard University, Cambridge, MA, USA). The tumor was independently identified by two individuals with a neurosurgery background. Three-dimensional models were constructed using the tumor outlines to determine the VS volume and VS surface area with the “Fast Marching” method in a 3D slicer software (version 5.4.0.; https://www.slicer.org (accessed on 24 November 2023)) [18]. The tumor shape was quantified using elongation (see Figure 1). The elongation is calculated from the square root of the ratio of the second largest principal moment to the second smallest: $Elongation=\sqrt{\frac{\lambda minor}{\lambda major}}$ [19].

### 2.3. Surgical Procedure and Follow-Up Regime

All the patients underwent tumor treatment in the semi-sitting position via the retrosigmoid approach. During the surgical resection, the patients were under general anesthesia. The access planning, resection limits, and tumor identification were guided by neuronavigation (Brainlab Curve, BrainLAB AG, Feldkirchen, Germany). First, a linear skin incision behind the ear was made to expose the connection between the transverse sinus and the sigmoid sinus by craniotomy. Throughout the procedure of slowly prepping between the cerebellar hemisphere and petrous bone to reach the internal acoustic meatus and resect the tumor, an intraoperative electromyogram (EMG) was used. The monitoring of the facial nerve was secured by the EMG recordings of the orbicularis oculi and oris muscles as well as electric stimulation. At the CPA, tumor debulking is performed with an ultrasonic surgical aspirator. Prior to this, the tumor surface is mapped with an electrophysiological probe to identify any rare dorsal positioning of the facial nerve, which is found in 0.6% of the cases [20]. Typically, the facial nerve runs ventrally along the VS. Once the VS tumor mass is sufficiently reduced, the cleavage plane between the tumor and surrounding arachnoid is dissected using bimanual techniques. The arachnoid and perineurium layers are gently separated from the VS as it is gradually mobilized and further reduced in size. Critical structures, such as the lower cranial and cochlear nerves, are carefully identified and preserved. The facial nerve is usually located medially at the brainstem exit zone, identified through direct electrostimulation, although its path can vary in giant VSs, necessitating a cautious, step-by-step dissection. Special care is taken in the juxta-meatal location to prevent crossing the point of facial nerve adhesion. In the cases of strong tumor adherence, a small remnant may be left to protect the integrity of the facial nerve. Bipolar coagulation is used sparingly, particularly near cranial nerves, and bleeding is controlled with targeted measures. The first clinical and follow-up appointment with MR images took place 3 months after surgery. Because of no visual neuroradiological progression of tumor growth or worsening of neurological deficits, follow-ups remain annually.

### 2.4. Immunohistochemistry

The material which was extracted during surgery was stained with hematoxylin/eosin. Molecular Immunohistology Borstel-I Antibodies (MIB1; DAKO, Glastrop, Denmark) were used on the sections to perform an immunohistochemical reaction to detect the Ki67 antigen. To determine the MIB-1 index, randomly selected high-power microscopic fields were analyzed for stained and unstained nuclei in the tumor cells. The percentage of Ki-67-positive nuclei defined the MIB-1 index [21].

### 2.5. Statistics of Institutional Data

Preoperative demographics, pre- and postoperative tumor volumes, and the extent of resection, radiomic tumor shape features, histopathological features, and immunohistochemical characteristics were compared in the patients with good facial nerve outcomes (FNOs) and poor FNOs using Fisher’s exact test (two-sided) for categorical data and independent t-test for continuous data. The optimum cut-off values of continuous data (elongation, age, extent of resection, and tumor volume) were determined using receiver-operating characteristics curves (ROC). A *p*-value < 0.05 was defined as statistically significant. 

A multivariable logistic regression analysis with age and sex adjustment was conducted to evaluate the predictors of FNO at 3 months after surgery. Tumor volume and the extent of resection were included due to the established role of these factors influencing facial nerve functioning [2].

## 3. Results

### 3.1. Patient Characteristics

The data of the 61 patients who met the inclusion criteria were analyzed. The median age (±SD) was 57 years (±15.39). The median KPS (interquartile range (IQR)) was 90. Preoperative facial nerve dysfunction was recorded in three patients. Two (3.3%) of them with a House–Brackman score of 3 and one (1.6%) patient with a House–Brackman score of 4. Preoperative hydrocephalus was present in two (3.3%) patients. Ipsilateral anacusis was present in 13 (21%) patients before surgery. 

The mean volume (±SD) and surface area (±SD) of the VSs were 9.95 cm^3^ ± 12.8 cm^3^ and 2434.46 mm^2^ ± 2173.79 mm^2^. In the study, only Koos grade 3 and Koos grade 4 patients were included, which were 23 (37.7%) and 38 (62.3%) patients for the higher grade. The mean (±SD) MIB-1 labeling index was 2.83 ± 1.23. Additional details are provided in Table 1.

### 3.2. Patient Characteristics in Good and Poor Facial Nerve Outcome

Good FNO was present in 50 cases (82.0%), and poor FNO was observed in 11 cases (18.0%) directly after surgery, respectively. After 3, 12, and 24 months, good FNOs were observed in 50 (82%), 44 (91.7%), and 43 (91.5%) patients, respectively, while poor FNO was found in 11 (18.0%), 4 (8.4%), and 4 (8.5%) cases in the control time phrases. The median age (±SD) at diagnosis was 53.73 ± 15.34 years for the patients with good FNO and 62 ± 14.33 years for those with poor FNO (*p* = 0.096). KPS and female sex were homogeneously distributed between the two groups. There was no significant difference in the mean surface area between the groups. Tumor volume (mean (±SD) tumor volume: 10 ± 13.7 cm^3^ (good FNO) vs. 9.6 ± 8.4 cm^3^ (poor FNO); *p* = 0.91 and tumor size (tumor size (size of tumor pre-op axial (mean ± SD)) 32.1 ±30.5 mm (good FNO) vs. 29.5 ± 8.9 mm (poor FNO); *p* = 0.77) did not differ significantly between good and poor FNO. Tumor elongation was significantly higher in the patients with good FNOs (0.48 ± 0.2 vs. 0.36 ± 0.13, *p* = 0.047). Furthermore, near or GTR was significantly associated with poor FNO at 3 months after surgery. The patient characteristics for good and poor FNO are summarized in Table 2.

### 3.3. Association between Elongation and Facial Nerve Outcome

**Figure 2:**
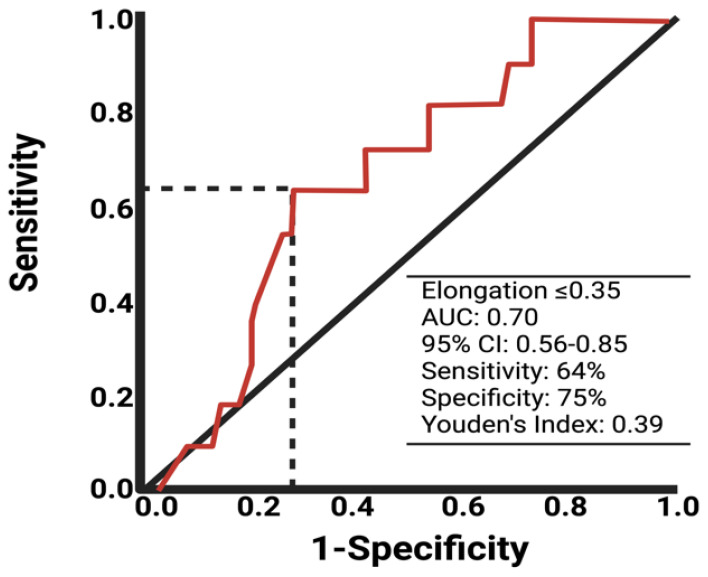
ROC curve of elongation in predicting poor facial nerve outcome after VS surgery.

Due to univariable significant differences in facial nerve outcomes regarding the shape parameter elongation and extent of resection, further analysis via the ROC curve was performed. The AUC of elongation in identifying the patients with a poor facial nerve outcome was 0.70 (95% CI: 0.56–0.85). The optimum cut-off value was identified at ≤/>0.35 with a sensitivity and specificity of 64.0% and 75.0%, respectively. Figure 2 shows the ROC analysis with the corresponding results. Cut-off value determination using the ROC curve analyses of the variables age, tumor volume, and extent of resection are provided in Supplementary Figure S1. For age (Supplementary Figure S1A), the optimal cut-off is ≥/<55 with a Youden’s index of 0.26, sensitivity of 46.0%, and specificity of 80.0%. For preoperative tumor volume (Supplementary Figure S1B), the cut-off is ≥/<2.6 cm^3^ with a Youden’s index of 0.22, sensitivity of 81.8%, and specificity of 40.0%. For the extent of resection (Supplementary Figure S1C), the cut-off is ≥/<93.4% with a Youden’s index of 0.44, sensitivity of 81.8%, and specificity of 62.0%.

**Figure 3:**
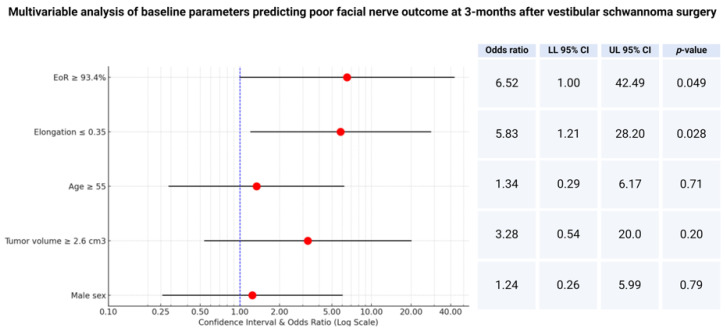
Forest plots from multivariable binary logistic regression analysis: extent of resection and elongation are independent predictors of poor facial nerve outcome at 3 months after surgery.

Therefore, a multivariable analysis of the factors potentially influencing facial nerve outcome has been performed. The multivariable logistic regression analysis included the extent of resection, baseline tumor volume, elongation, age, and sex. The multivariable analysis identified two factors independently predicting poor facial nerve outcome at 3 months after VS surgery: (1) extent of resection ≥ 93.4% (OR: 6.5; 95% CI: 1.0–42.5; *p* = 0.049) and (2) elongation ≤ 0.35 (OR: 5.8; 95% CI: 1.2–28.2; *p* = 0.028). Figure 3 shows the forest plots summarizing the multivariable analysis.

### 3.4. Association between Shape and Proliferation

**Figure 4:**
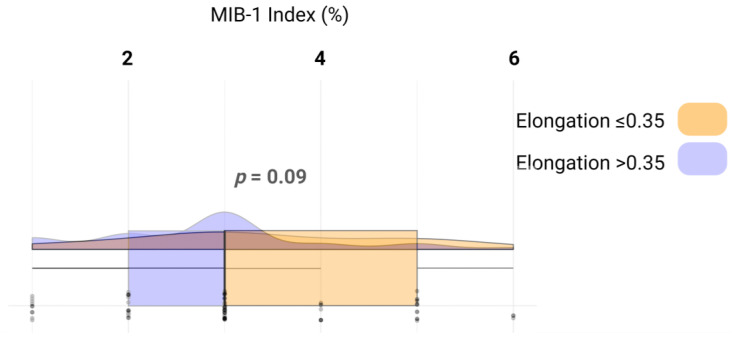
Raincloud plots, box plots, and raw data points illustrate MIB-1 labeling indices among those with an elongation ≤ 0.35 (yellow) and those with an elongation > 0.35 (blue).

We analyzed whether the increased proliferation reflected by the MIB-1 labeling index influences the elongation of the VSs. The mean MIB-1 labeling index in those with an elongation ≤ 0.35 was 3.4 +/− 1.4, whereas in those with an elongation >0.35 was 2.8 +/− 1.2 (independent t-test: *p* = 0.09). Figure 4 illustrates the relevant metrics and distribution of the data.

## 4. Discussion

This study investigated the impact of tumor elongation on the facial nerve outcome after surgery in the semi-sitting position via the retrosigmoid approach. We examined the facial nerve outcomes of 61 patients undergoing surgery at our institution and found a significant impact of elongation on the postsurgery facial nerve outcome. The shape of the tumor seems to have a high impact on surgery outcomes.

The existing literature suggests that a larger tumor size significantly increases the risk of poor facial nerve outcomes, both anatomically and functionally [2,22]. According to the majority of the reported studies, facial nerve function was only preserved in 27–58% of cases after the total microsurgery resection of large VS (tumor size over 3 cm) [22,23,24,25,26,27,28]. 

Currently, most of the tumor centers prefer an STR against an NTR or GTR [8,29]. Studies show that the facial nerve outcome 1 year after surgery is higher when the tumor remains inside the patient [11]. In the meta-analysis published by Gurgel et al. [9], 92.5% of the patients who underwent STR had a good facial nerve outcome compared to the 47.3% who underwent GTR. 

Hence, it appears to be essential to protect the facial nerve in its course during the surgery rather than perform a complete resection. Given the benign nature and slow cell proliferation rate of these tumors, it is crucial to prioritize patient’s quality of life. On the other hand, Vakilian et al. [30] found that all VS with a postsurgical volume greater than 2.5 cm^3^ recurred. In these cases, further surgery or a different treatment like gamma knife radiosurgery is needed. It seems beneficial to initiate postoperative stereotactic radiosurgery in subtotal resected VS within 1 year after surgery [3,14,28]. A study by Pan et al. [31] compared two groups of patients with large VS. The first one underwent intracapsular decompression, followed by gamma knife surgery, while the second group underwent a total resection, also followed by gamma knife surgery. They found a good facial nerve outcome (HB1-2) in 89% in group one and only 35% in group two. A study by Strickland et al. [32] is questioning these results. They found superior tumor control and a higher likelihood of facial nerve recovery in NTR compared with STR. While in the immediate postoperative period, 50% of the NTR and 52% of the STR had good facial nerve function, in the long-term follow-up, 76.9% of the NTR and only 56% of the STR had no or mild dysfunctions. In line with various studies [8,30,33,34,35], they found a 3–12-fold higher regrowth risk and a median time of recurrence that is three times shorter following STR compared to NTR. However, our study also showed a significant association with the extent of resection. An extent of resection ≥ 93.4% seems to be a risk factor for poor facial nerve outcome. A study by Zhang et al. [36] came to similar results. They found good facial nerve outcome and long-term tumor control of 58.6% and 96.6% in GTR, 79.6% and 92.2% in NTR, and 83.3% and 76.2% in STR, respectively. NTR combines the good facial nerve outcome similar to STR and a similar tumor-free regrowth rate to GTR. There is an ongoing discussion between surgeons concerning GRT or STR. Currently, gamma knife surgery has gained popularity and shows excellent results in tumor growth control and preventing the facial and vestibular nerve [37,38,39,40,41]. The tumor growth control rate varies for large VS from 54% to even 100% [22,39,42,43,44,45,46,47,48,49,50]. A recent study by Roethlisberger et al. [51] analyzed 48 patients with Koos grade IV vestibular schwannomas who underwent STR followed by a “wait-and-scan” protocol and found that 81% of the tumor residuals were stable or regressed over 4.4 years. Tumor progression occurred in 19% of the cases, with higher postoperative volumes linked to greater progression risk. Second-line radiation therapy (RT) was needed in 29% of the patients, achieving a 96% overall tumor control rate, with no cases requiring salvage surgery, highlighting the effectiveness of STR combined with targeted RT for long-term tumor control. Further studies are needed to investigate if an STR followed by gamma knife surgery can benefit a good facial nerve outcome while maintaining a low regrowth rate. 

In contrast to the previously mentioned studies [14,52,53], we did not find a significant correlation between the tumor size and facial nerve outcome. Nevertheless, we identified that the tumor shape reflected by the parameter elongation is of importance. We observed that a more elongated tumor shape led to a poorer facial nerve outcome (*p* = 0.047) at 3 months after VS surgery. From the perspective of the anatomical conditions, it might be plausible that an elongated tumor with an increased intra-meatal tumor portion has a higher risk of damaging the facial nerve in its course. The facial nerve exits the brainstem in the area of the cerebellopontine angle. From there, it pulls through the internal auditory canal together with the vestibulocochlear nerve. Therefore, the longer the vestibular schwannoma is, the longer there is a close course between the two nerves, and the higher the risk of damaging the facial nerve during surgery. From a pathophysiological point of view, we suggest that a more elongated tumor shape might be the result of heterogeneity in proliferative potential in various areas of VSs, with some hotspots exhibiting significantly increased growth rates. We found a tendency (*p* = 0.09) for increased MIB-1 labeling indices among those with more elongated VSs and assume that proliferative heterogeneity significantly impacts tumor shape and facial nerve outcome. However, the degree of tumor-associated macrophage infiltrates overlapping with tumor cells in the present VS series remains unknown and might also impact these endpoints [7]. The anatomic circumstances need to be considered when planning the operational access. Currently, there are mainly two ways of positioning the patient that are discussed. A study by Roessler et al. [54] investigated the postoperative facial nerve function in lateral positioning vs. semi-sitting positioning in medial-sized to large VS. They found a significantly lower rate of facial palsies and hearing loss in the semi-sitting positioning while no higher risk of surgical complication rate like venous air embolism. In 63%, no facial palsy was detected in the semi-sitting positioning group compared to only 40% in the lateral positioning group. The study of Schackert et al. [55] came to similar conclusions. Here, facial nerve function was retained in 81.2% via the semi-sitting positioning vs. 74.5% during lateral positioning. Due to the small or even negative venous pressure in a sitting position, the cerebellum sinks down and widens the room for the surgeon, who now has easier access to the cerebellum pontine angle. Nevertheless, the distance the surgeon needs to separate the tumor from the nerves correlates with the elongation of the tumor. Despite the better access to the tumor or the size of the whole tumor, the risk of damaging the facial nerve increases with the elongation of the tumor which needs to be dissected from the nerve. This contact time seems to be a huge risk for facial nerve damage. A well-trained surgeon and good intraoperative electromyogram monitoring and intraoperative facial nerve monitoring (IOFM) can promote the operating result [14]. The retrospective study design and the different surgical skills of the surgeons are the major confounding factors of our analysis. Further studies are needed to investigate if the training of the surgeon affects the facial nerve outcome.

The major limitation of this study is its retrospective and monocentric design. The surgical dissection of the facial nerve in giant VS can be challenging and it might be possible that this factor also influences the statistical results despite a homogeneous institutional surgical approach. Moreover, this study has a small number of patients and does not include EMG data during the surgery, which might reveal a better insight into the amount of facial nerve irritation during surgery. However, we created a homogenous collective of patients applying highly selective inclusion criteria. All the patients underwent surgery using the semi-sitting position via a retrosigmoid approach and had only sporadic VS, which limited the external influences on the treatment. 

## 5. Conclusions

The present study shows that in Koos grade 3 and 4 tumors, elongated VS shape seems to be a risk factor for worsened facial nerve outcome at 3 months after surgery. In the case of an elongated tumor shape in VS, an NTR might be safer to secure the facial nerve function against a GTR. 

## Figures and Tables

**Table 1:** Patient characteristics.

Characteristics	N = 61
Median age (years, ±SD)	57 (15.39)
Female sex	32 (57.4%)
Median preoperative KPS (IQR)	90 (50–100)
Dysphagia preoperative	4 (6.6%)
Preoperative CN VII dysfunction	
HB II	2 (3.3%)
HB III	0
HB IV	1 (1.6%)
Hydrocephalus preoperative	2 (3.3%)
Arterial HT	32 (52.5%)
Surface area, (mean ± SD), cm^2^	24.34 ± 21.74
Tumor volume, (mean ± SD), cm^3^	9.94 ± 12.8
Tumor size preoperative (cm) axial (mean ± SD)	3.16 ± 2.8
Flatness	1.22 ± 0.11
Roundness	0.83 ± 0.06
Elongation	0.46 ± 0.19
MIB-1 index (mean ± SD)	3.0 ± 1.3
Koos Grade	
3	23 (37.7%)
4	38 (62.3%)

Abbreviations: CN = cranial nerve; KPS = Karnofsky Performance Status; MIB = Molecular Immunology Borstel; SD = standard deviation.

**Table 2:** Comparison of patient characteristics between good and poor FNO (using Fisher’s exact test (two-sided) and independent t-test).

Characteristics	Good FNO (n = 50)	Poor FNO (n = 11)	*p*-Value
Mean age (years +/− SD)	53.73 +/− 15.342	62.0 +/− 14.327	0.096
Male sex Female sex	22 (84.6%) 28 (80.0%)	4 (15.4%) 7 (20.0%)	0.75
Mean preoperative KPS (+/−SD)	90.0 +/− 8.08	89.1 +/− 8.31	0.74
Cystic VS	25 (50.0%)	8 (72.7%)	0.32
Surface area, (mean ± SD), cm^2^	24.20 +/− 22.87	24.92 +/− 17.18	0.919
Tumor volume, (mean ± SD), cm^3^	10.03 +/− 13.73	9.56 +/− 8.43	0.914
Tumor size (size of tumor preoperative (cm) axial (mean ± SD))	3.21 +/− 3.05	2.95 +/− 0.89	0.77
Elongation (+/−SD)	0.48 +/− 0.20	0.36 +/− 0.13	**0.047**
MIB-1 index, (mean ± SD)	3.0 +/− 1.3	2.8 +/− 1.3	0.61
Extent of resection			
GTR or NTR STR or partial resection	22 (71.0%) 28 (93.3%)	9 (29.0%) 2 (6.7%)	**0.04**

Abbreviations: KPS = Karnofsky Performance Status; MIB = Molecular Immunology Borstel; SD = standard deviation.

## Data Availability

All data are included in this manuscript.

## Supplementary Materials

The following are available online at https://www.mdpi.com/article/10.3390/jcm13175319/s1, Figure S1.
