# Supplementary figures and images for: The Impact of Tumor Elongation on Facial Nerve Outcome after Surgery for Koos Grade 3 and 4 Vestibular Schwannomas in the Semi-Sitting Position via the Retrosigmoid Approach
Source: J Clin Med. 2024 Sep 8;13(17):5319. doi: 10.3390/jcm13175319 (PMC11396018; doi:10.3390/jcm13175319)

**A**

## Age

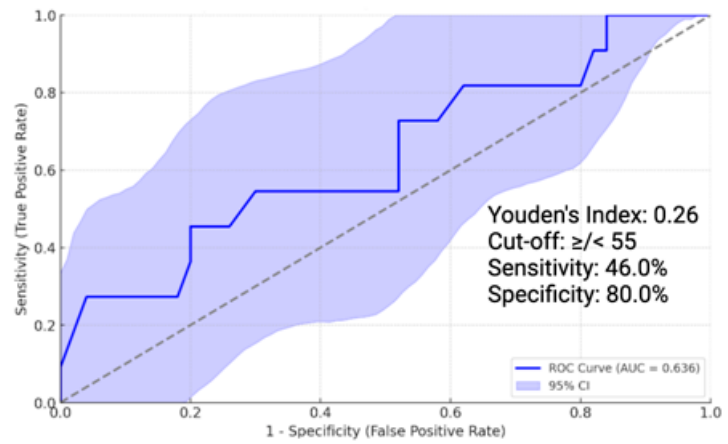**B**

## Preoperative tumor volume

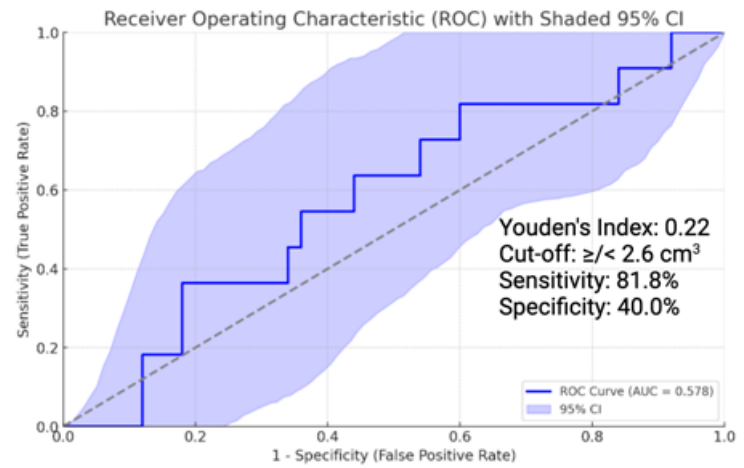**C**

## Extent of resection

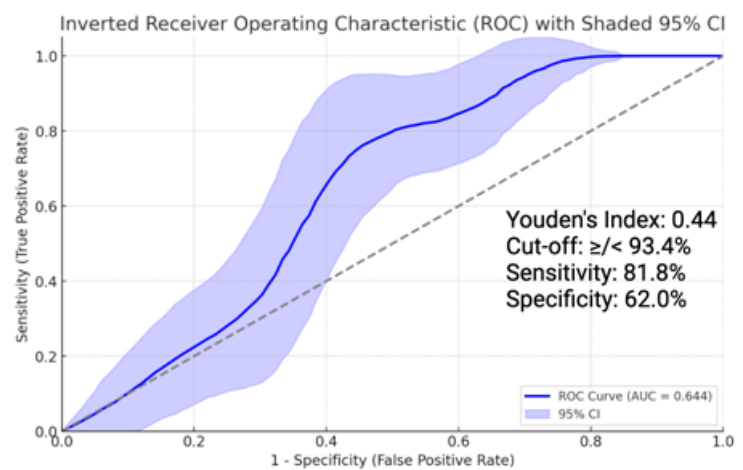

Figure S1.

Supplement: Supplementary file 1 [file jcm-13-05319-s001.zip › jcm-3174046-supplementary materials.pdf]
